# Editorial: Role of Microbes in Climate Smart Agriculture

**DOI:** 10.3389/fmicb.2019.02756

**Published:** 2019-11-26

**Authors:** Suvendu Das, Adrian Ho, Pil Joo Kim

**Affiliations:** ^1^Institute of Agriculture and Life Sciences, Gyeongsang National University, Jinju, South Korea; ^2^Institut für Mikrobiologie, Leibniz Universität Hannover, Hanover, Germany; ^3^Division of Applied Life Science, Gyeongsang National University, Jinju, South Korea

**Keywords:** sustainable agriculture, greenhouse gas emissions and mitigation, C transformation and stability, extreme weather events, elevated CO_2_ and O_3_

Soil microbes play an essential role in virtually all ecosystem processes, such that microbial abundance and activity determines the sustainable productivity of agricultural lands, ecosystem resilience against nutrient mining, degradation of soil and water resources, and GHG emissions (Wagg et al., 2014). Their activity is directly affected by changes in the environment. In this context, climate change is a relevant factor, with the potential to affect the role of microbes in the soil, which is vital to support agriculture worldwide. Climate-smart agriculture (CSA) is an approach that can help to reduce these impacts. CSA is an integrative approach to develop agricultural strategies for sustainably increasing agricultural productivity, adapting, and building resilience of agricultural and food security systems, and reducing agricultural greenhouse gas emissions under climate change scenarios (Lipper et al., 2014; Paustian et al., 2016). In this Research Topic, we aimed to provide the reader with a selection of studies to highlight novel experimental concepts such as process-oriented omics approaches with state-of-the-art technological advances in agricultural science to better understand how consequences of climate change such as elevated atmospheric CO_2_ concentration (eCO_2_), temperature, and drought affect soil microbes and associated ecosystem processes. In addition, the role of microbes in agricultural management that contribute to climate change adaptation, GHG mitigation, and soil carbon storage has been discussed.

As two core issues of global climate change, the constant rise in atmospheric CO_2_ concentration and temperature have significant influences on ecosystem functioning (Mueller et al., 2016). In a study in semiarid grassland ecosystems, Yu et al. revealed the potential feedback response mechanism of soil microbiome to multiple climate change factors by the decrease in N cycling processes under warming, and increase in C and N cycling processes under either eCO_2_ alone or in interaction with warming. In the context of increasing global atmospheric CO_2_ concentration, grasslands behave as a potential C sink (Roy et al., 2016). Clipping (removal of aboveground plant biomass) is a common practice in grassland ecosystems, and this practice may reduce nutrient inputs into soils (Garibaldi et al., 2007), which in turn may affect microbial functionality and by extension, other ecosystem services. Accordingly, Guo et al. concluded that annual clipping shifted functional communities and enhanced the relative abundance of genes related to labile and recalcitrant C degradation with potential links to a clipping-induced acceleration of decomposition of C stored in grassland ecosystems.

The impact of climate warming on soil C and N dynamics has recently received considerable interest. Waghmode et al. revealed that climate warming and dried soil conditions remarkably increased the abundance of ammonia-oxidizing bacterial (AOB), concomitant to a reduction in the abundance of ammonia-oxidizing archaea and denitrifying bacteria, potentially affecting nitrogen turnover in the agro-ecosystem. The authors further suggested that, compared to regular irrigation (60 mm), the high irrigation (90 mm) overrode the warming effects on soil microbial community structure. The effects of extreme weather events on pathogen-antagonist interactions were evaluated in a perspective article by Meisner and de Boer. Extreme weather events like droughts or heavy precipitation are becoming more frequent and affect agricultural ecosystems (e.g., plant health and productivity). Soil-borne plant pathogens might become a bigger problem if microbial antagonists in soils are more strongly affected by the extreme weather conditions than the pathogens and can thus not suppress pathogens in soils. Different strategies of microorganisms to cope with water stress were discussed, and the potential for controlling soil-borne plant pathogens through enhancing growth of beneficial microorganisms under extreme weather conditions was highlighted in the perspective.

CSA emphasizes developing agricultural strategies not only to protect food security under climate change but also to mitigate GHG emissions and to improve soil C sequestration (Lipper et al., 2014). Biochar (the C-rich solid formed by pyrolysis of biomass) amendment in agricultural soil has been proposed as a way to abate climate change by sequestering C and mitigating GHG (particularly N_2_O), while simultaneously increasing the crop yield (Woolf et al., 2010; Jiang et al., 2019). In an innovative research, Wang et al. revealed that the biochar predominately reduces CH_4_ and N_2_O emissions with high straw load, but not with low straw load, and this could be because biochar competes for electrons against methanogens and promotes methanotrophs, nitrifiers and denitrifiers. Agricultural intensification results in the enhanced re-investment of bio-based residues in agricultural soils, with consequences for GHG emissions (Ho et al., 2017). In this contest, Brenzinger et al. suggested that the combination of compost with one of the more nutrient-rich organic amendments such as sewage sludge digestate provides a trade-off between sustaining crop yield and reducing GHG emissions. Duan et al. documented that the application of catch crop residues leads to higher N_2_O emissions, which could be due to net N mineralization and O_2_ depletion coupled with the residue degradation in organic hotspots. The catch crop residue amendment can influence the N_2_O production, but not the genetic potential of the community to produce and reduce N_2_O. Further, Mohanty et al. advocated that biogenic nitrate and microbial volatile organic compounds (mVOCs) could have positive feedback effects on the nitrification rate in arable soils. To this end, Norton and Ouyang reviewed the status quo of the controlling factors and management practices of soil nitrification. Management strategies to reduce N losses, improve N use efficiency, and mitigate global climate change were recommended based on the latest understanding of the nitrification process.

The Research Topic further focused on the potential use of slag (byproducts generated during iron and steel manufacturing) fertilizer for sustainable agricultural production. Iron and steel production rose dramatically with the advent of the industrial revolution, and the volume of slag produced outpaced its consumption. Slags are rich in fertilizer components and their use in agriculture holds great promise for sustainable and eco-friendly agriculture (Gwon et al., 2018; Das et al., 2019). In a mini-review, Das et al. discussed the potential mechanisms of slag-microbe interactions in soil and how the interactions influence crop yield, GHG emissions, soil carbon sequestration, and heavy metal stabilization in contaminated soils.

CSA also emphasizes the sustainable development of livestock manure production for mitigating CH_4_ emissions, since livestock production is a significant source of methane, mainly from enteric fermentation, dairy farming operations, and manure management (Laubach et al., 2015). In a study, Habtewold et al. concluded that the acidified dairy slurry suppressed CH_4_ emissions, which could be due to the inhibition of *Methanosarcina* species.

The need for increased food production under CSA interventions increasingly shifted the focus to the role of soil biodiversity in general and arbuscular mycorrhizal (AM) fungi in particular. In a review, Sosa-Hernández et al. presented an overview on the current knowledge of subsoil ecology with the focus on arbuscular mycorrhizal fungi (AMF) and their potential significance for a sustainable agriculture. Practices of no-tillage, crop rotations, and cover cropping with deep rooting mycorrhizal plants may promote subsoil AM communities.

A deep understanding of microbial ecology and soil–plant–microbe interactions in a changing climate scenario is essential to use microbial technology for climate change adaptation and mitigation. This Research Topic contributes to the understanding of how climate changes affect soil microbes and ecosystem processes, and how agricultural practices under CSA interventions shifted microbiome for climate change adaptation, GHG mitigation, and soil C storage.

## Author Contributions

SD wrote the first draft of the editorial. All authors edited the editorial.

### Conflict of Interest

The authors declare that the research was conducted in the absence of any commercial or financial relationships that could be construed as a potential conflict of interest.
